# External Quality Assurance of Malaria Nucleic Acid Testing for Clinical Trials and Eradication Surveillance

**DOI:** 10.1371/journal.pone.0097398

**Published:** 2014-05-16

**Authors:** Sean C. Murphy, Cornelus C. Hermsen, Alexander D. Douglas, Nick J. Edwards, Ines Petersen, Gary A. Fahle, Matthew Adams, Andrea A. Berry, Zachary P. Billman, Sarah C. Gilbert, Matthew B. Laurens, Odile Leroy, Kristen E. Lyke, Christopher V. Plowe, Annette M. Seilie, Kathleen A. Strauss, Karina Teelen, Adrian V. S. Hill, Robert W. Sauerwein

**Affiliations:** 1 Department of Laboratory Medicine and Center for Emerging and Re-Emerging Infectious Diseases, University of Washington (UW), Seattle, Washington, United States of America; 2 Department of Medical Microbiology, Radboud University Medical Center (RUMC), Nijmegen, The Netherlands; 3 The Jenner Institute, University of Oxford, Oxford, United Kingdom; 4 European Vaccine Initiative (EVI), Heidelberg, Germany; 5 Department of Laboratory Medicine, Clinical Center, National Institutes of Health (NIH), Bethesda, Maryland, United States of America; 6 Center for Vaccine Development and Howard Hughes Medical Institute, University of Maryland School of Medicine, Baltimore, Maryland, United States of America; Food and Drug Administration, United States of America

## Abstract

Nucleic acid testing (NAT) for malaria parasites is an increasingly recommended diagnostic endpoint in clinical trials of vaccine and drug candidates and is also important in surveillance of malaria control and elimination efforts. A variety of reported NAT assays have been described, yet no formal external quality assurance (EQA) program provides validation for the assays in use. Here, we report results of an EQA exercise for malaria NAT assays. Among five centers conducting controlled human malaria infection trials, all centers achieved 100% specificity and demonstrated limits of detection consistent with each laboratory's pre-stated expectations. Quantitative bias of reported results compared to expected results was generally <0.5 log_10_ parasites/mL except for one laboratory where the EQA effort identified likely reasons for a general quantitative shift. The within-laboratory variation for all assays was low at <10% coefficient of variation across a range of parasite densities. Based on this study, we propose to create a Molecular Malaria Quality Assessment program that fulfills the need for EQA of malaria NAT assays worldwide.

## Introduction

During the past two decades, numerous nucleic acid testing (NAT) approaches for the diagnosis of human malaria infection have been developed [1]–[21]. NAT can detect and quantify parasites more sensitively and precisely than by microscopy or rapid diagnostic tests (RDTs). NAT approaches are valuable for controlled human malaria infection (CHMI) studies of investigational drug and vaccine candidates, for drug efficacy studies and for epidemiological surveillance [22]. In CHMI studies for example, healthy human volunteers are infected via the bites of *Plasmodium falciparum*-infected mosquitoes [23]–[25] or by needle-based delivery of purified sporozoites [26], [27]. CHMI is used in initial efficacy studies of investigational drugs and vaccines because of its reproducibility and convenience as compared with efficacy studies in malaria-endemic populations that require larger studies and rely on natural exposure [26], [28]–[40]. NAT assays allow for quantitative measurement of peripheral parasitemia up to 2–6 days earlier than microscopy [26], [27], [30], [41], further improving the safety of this already very safe model. Depending on the trial design and the laboratory capabilities, samples can be tested either in real time using fresh samples or retrospectively using archived samples. NAT is less operator-dependent and more amenable to high throughput testing than microscopy but is more expensive. The increased cost of NA testing in clinical trials affords improved discrimination between infected and uninfected subjects (e.g., fewer false positives and false negatives) and production of quantitative datasets that can be used for modeling parasite growth. In addition, because earlier detection and therefore earlier treatment of asymptomatic parasitemia decreases both volunteer risk and discomfort, use of NAT assays can facilitate the elimination of the costly but traditional ‘hotel’ phase of many studies where volunteers are housed near study staff for close monitoring. The many advantages and disadvantages of microscopy, RDTs and NAT vary depending on whether the result is used for monitoring of clinical trials, for clinical care in endemic or non-endemic settings or for epidemiological surveillance, as recently reviewed [22].

When testing is performed on the day of collection, NAT results are used in some centers alongside clinical assessments and microscopic findings to inform treatment decisions. This is useful since the symptoms of malaria are non-specific and overlap with common viral illnesses. NAT results can be particularly useful when these traditional measures like microscopy are inconclusive. Despite use of highly-trained slide readers, detection of 1–2 parasites on a thin blood smear is neither 100% sensitive nor specific for the low parasite density capable of causing symptomatic malaria. Some CHMI centers have even replaced primary microscopy-based endpoints with primary NAT endpoints. When NAT was performed in real time and used to make treatment decisions, this approach reduced reported clinical symptoms in CHMI subjects relative to microscopy-based treatment decision making (G. Bastiaens, unpublished data). Thus, the NAT result can help eliminate diagnostic uncertainly that may otherwise occur in subjects who are either ostensibly slide positive or symptomatic but not both. Overall, real time availability of NAT apparently helps avoid false positive and false negative diagnoses, thereby increasing both the safety of the volunteers and the accuracy of the data. Aside from lower limits of detection (LoD) that afford earlier diagnoses, quantitative NAT data provide day-by-day measures of the rise and fall of parasitemia and allow for model-based assessments of liver-to-blood inocula levels and parasite multiplication rates, which can be used to calculate efficacy estimates for partially-effective liver vaccines and for blood-stage vaccines [42], [43].

Quality systems are critical to clinical trial and surveillance networks because the validity of diagnostic and monitoring tests and the ability to compare trial results amongst network laboratories is entirely dependent on the procedures used before, during and after each assay at each site [22]. Consistently dependable results are provided when the overall program includes quality control (QC), quality assurance (QA) and proficiency testing (PT) [44]. Malaria QA systems have been developed to address the wide performance variations observed in first generation RDTs [45]. These efforts resulted in consistently improved RDT performance with each successive round of evaluations and led to elimination of poor RDT products. External QA (EQA) programs and control reagents for RDTs are also emerging [46]–[48]. Terminology related to quality management and assay performance is defined in the Glossary section.

Because of the availability of cryopreserved metabolically-active, non-replicating sporozoites [49] and the renewed vigor of vaccine and drug pipelines, CHMI studies are now being conducted at an increasing number of institutions worldwide [50]. However, while consensus procedures for CHMI studies and microscopy are available [51], no such effort has been made to standardize malaria NAT assays or provide widespread ongoing EQA oversight. Some laboratories have exchanged small panels of malaria-infected whole blood with collaborators as part of NAT assay validation (S. Murphy, C. Hermsen, N. Edwards, A. Stewart, unpublished data). In addition, the World Health Organization (WHO) previously generated freeze-dried *P. falciparum*-infected blood samples at a single high parasitemia level for use as an international DNA standard [52]. Some laboratories are using the WHO material (for example see reference [53]), but the WHO material is not provided through a formal EQA program. Because the material was freeze-dried from whole blood without buffers or steps to protect RNA, it also cannot be used to support RNA-based assays, and the freeze-dried material does not precisely mimic material obtained from clinical trial participants. Therefore, a formalized, funded program is needed to ensure assay validation and provide malaria NAT EQA in line with that commonly used for assays detecting HIV and other infectious pathogens [54]–[56]. As a trial EQA platform, we sent blinded specimens to five malaria centers conducting CHMI studies. Here, we report the results of this exercise and propose a framework for ongoing EQA and eventually for assay harmonization.

## Materials and Methods

### Malaria culture and production of samples

*P. falciparum* strain 3D7 was cultured, synchronized and diluted as previously reported [41]. For multiply-infected cells, each parasite was counted in microscopic parasite density measurements. A ring-stage synchronous high parasitemia culture was diluted into type A+ whole human blood obtained from the Puget Sound Blood Center (www.psbc.org). After preparing the ‘master’ tube at each density, samples were aliquoted into bar code-labeled tubes [each bearing a unique specimen identifier generated in the Laboratory Data Management System (Frontier Science)] and prepared for frozen storage according to the individual standard operating procedures for each final testing laboratory. Aliquot sizes were as follows: RUMC and University of Maryland 0.5 mL; NIH 0.2 mL into 2 mL NucliSENS lysis buffer (bioMérieux); University of Washington (UW) 0.05 mL into 2 mL NucliSENS lysis buffer; Oxford filtered to remove leukocytes as described [57] and aliquoted as 0.5 mL volumes. Once aliquoted, all samples were frozen at −80°C before courier shipments to partner laboratories on dry ice.

### Sample testing

Laboratories received and stored samples at ≤−70°C before testing. Each lab except for the NIH received 10 de-identified parasite-containing samples at each of five different concentrations plus 10 parasite-negative samples; the NIH received 20 samples at each level. Labs were blinded to the parasite concentration in each sample. Each laboratory tested their designated samples according to the laboratory-specific standard operating procedure (SOP) and reported data to the UW coordinating center. Testing laboratories (including the technologists at the UW coordinating laboratory) were blinded to the nominal parasite density of each sample. Assays used included *P. falciparum* quantitative reverse transcription polymerase chain reaction (qRT-PCR; UW, [58]); quantitative PCR (qPCR; RUMC, [21], [59]; Oxford [26]; University of Maryland [35], [37]) and standard PCR (NIH [34]). If known, laboratories indicated their in-house determined limits of detection (LoD) and nucleic acid target characteristics (**Table 1**). All laboratories reported quantitative data, except the NIH which reported qualitative results and cycle thresholds (C_T_) from standard PCR. Each laboratory was asked to run the assay and provide data strictly in accordance with the SOP used in their clinical trials.

**Table 1:** Reported characteristics and use of network assays.

	Test characteristics				Used in CHMI?^c^	
Site	Assay method	Blood volume (µL)	Expected LoD^a^ (log_10_)	18S target^b^	During trial	After trial
**UW**	Automated extraction + qRT-PCR [58]	50	20 (1.30)	A-type rRNA & rDNA		[41], [58] - NCT01058226 & NCT01500980
**Oxford**	Leukocyte filter, manual extraction + qPCR [26]	500	10 (1.00)	S-type rDNA	[26] - NCT01465048; [38] - NCT00890760; [39] - NCT01142765; [40] - NCT00984763; NCT01623557*; NCT01883609*	NCT01666925; NCT01658696; NCT01379430
**NIH**	Semi-automated extraction + PCR [34]	200	ND (ND)	A-type rDNA	[34] (NCT01546389)	
**RUMC**	Semi-automated extraction + qPCR [21], [59]	500	20 (1.30)	S-type rDNA	NCT01728701**	[24], [27], [72] - NCT00442377 & NCT00757887; [25] -NCT01236612; [27] - NCT01086917; [33] - NCT01031524; [73] - NCT00870987; [74] - NCT00385047; [32] - NCT01627951; [21] - 0004-0090; [75] - 0011-0262; [76] - 2001/203, 2002/170; NCT01660854*; NCT01218893*; NCT01422954*; NCT01540903*; NCT01627951*; NCT00509158*; NCT01988636*; NCT01783340**
**Maryland**	Manual extraction + qPCR [35], [37]	500	40 (1.60)	S-type rDNA	[35], [37] - NCT00744133; [77] - NCT01001650; NCT01546389*	[34] - NCT01441167

a LoD, limit of detection in parasites/mL as independently determined by each laboratory before this EQA project.

b A-type  =  asexual-stage expressed 18S rRNA; S-type  =  sexual-stage expressed 18S rRNA; rRNA  =  RNA target; rDNA  =  coding gene target.

c When available, published references are listed to indicate the performance of NAT either during or after the listed trials. For studies completed but not yet published (*) and/or upcoming (**), the identifier from ClinicalTrials.gov (alphanumeric) or the Dutch Ethical Committee (numeric) is listed.

### Filtration studies (Oxford only)

Ring-stage cultured 3D7 strain *P. falciparum* parasites were added to leukocyte-depleted [26] whole blood. Each sample was subsequently divided and half of the material at each density was subjected to Whatman VFE filtration while half remained unfiltered. The material was then subjected to DNA extraction and qPCR by the standard Oxford protocol [26].

### Calibrator matrix studies (Oxford only)

DNA was extracted from blood from malaria-negative volunteers using the standard Oxford protocol [26] to generate ‘negative blood matrix' samples. qPCR was performed on 150 copies of the Oxford plasmid DNA calibrator in the presence of a blood matrix sample or a water control (20% v/v) and differences in the C_T_ and quantity were evaluated.

### Data analysis

Data were transformed to log_10_ parasites/mL of whole blood and analyzed using Excel 2010 (Microsoft) and Prism 6 (GraphPad). Intra-laboratory performance was evaluated using sensitivity/specificity analyses, precision analyses and Bland-Altman (difference) plots. The lowest parasite density samples (6 parasites/mL) were not included in statistical analyses. Data were plotted on a log_10_ parasites/mL scale; data from the NIH was regressed to the nominal values of the provided samples and plotted for illustration purposes. For precision studies, the percent coefficient of variation was calculated as %CV  =  standard deviation/mean.

## Results

### Sample production

A high parasitemia starting culture was prepared to 2.4×10^8^ (8.38 log_10_) parasites/mL based on repeated counts of Giemsa-stained thin blood smears by multiple readers combined with determination of RBC density (RBC/mL) by hemocytometer counting. This material was devoid of trophozoite- and schizont-stage parasites and contained singly (70%) and multiply-infected (30%) ring-stage parasites as judged by microscopy. Dilutions were made into whole blood to nominal parasite densities as follows: High (300,000 parasites/mL or 5.48 log_10_ parasites/mL); Mid (6,000 parasites/mL or 3.78 log_10_ parasites/mL); Low (600 parasites/mL or 2.78 log_10_ parasites/mL); Very Low (60 parasites/mL or 1.78 log_10_ parasites/mL), Trace (6 parasites/mL or 0.78 log_10_ parasites/mL) and Negative (no parasites); these designations are used throughout the paper to refer to these specific parasite densities.

### Network laboratory results

**Figure 1:**
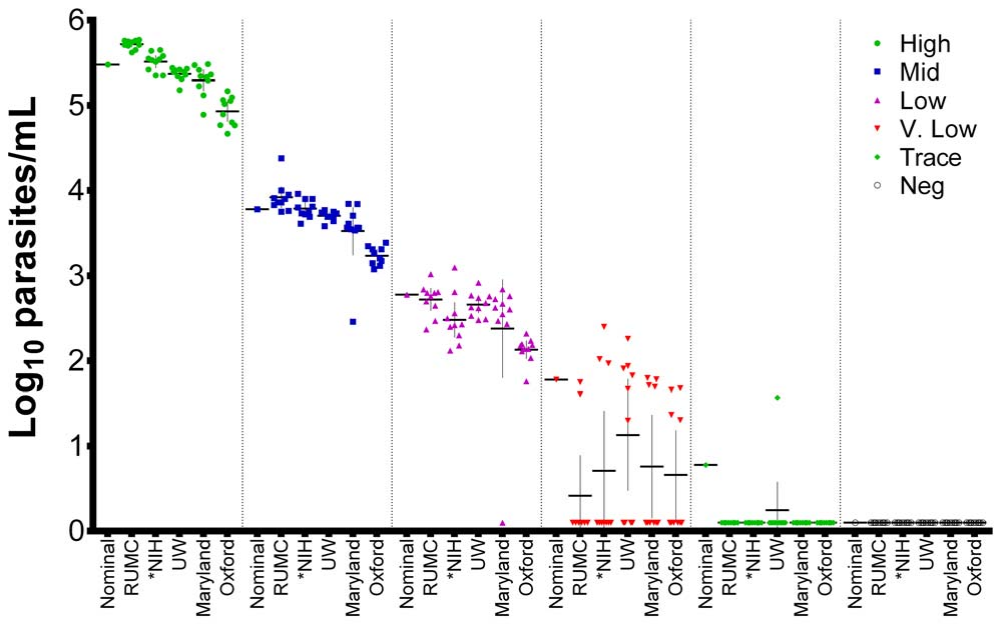
Study data. Results were plotted on a log_10_ parasites/mL scale for the five participating laboratories; bars show the mean and 95% confidence interval. Nominal (expected) values for all samples are plotted as follows: high (300,000 parasites/mL or 5.48 log_10_ parasites/mL); mid (6,000 parasites/mL or 3.78 log_10_ parasites/mL); low (600 parasites/mL or 2.78 log_10_ parasites/mL); very low (60 parasites/mL or 1.78 log_10_ parasites/mL), trace (6 parasites/mL or 0.78 log_10_ parasites/mL) and negative (no parasites). Samples with no parasites detected were plotted as 0.1 log_10_ parasites/mL. Two-way ANOVA comparisons across all high, mid and low parasite density samples with quantitatively positive results showed non-statistically significant differences amongst all groups (*p*>0.05) except for RUMC vs. Oxford at high (*p*<0.0001), mid (*p*<0.01) and low (*p*≤0.05) parasite densities. *NIH quantities were generated by regression of C_T_ values to expected EQA values and are provided to visualize variation and qualitative agreement; quantitative statistical comparisons were not included. 60 of 120 representative NIH samples are displayed for consistency.

Laboratories reported the results shown in the **Figure 1**. The sensitivity and specificity of each laboratory's assay was calculated based on the negative control samples plus either all samples ≥60 parasites/mL or those ≥600 parasites/mL; samples at 6 parasites/mL were not included in these analyses since this ultra-low level of parasite density was below the stated LoD for all assays (**Table 2**). All laboratories demonstrated 100% specificity. Most laboratories detected all samples at ≥600 parasites/mL, although the University of Maryland reported one false negative at this level.

**Table 2:** Sensitivity and specificity by laboratory.

	Excluding 6 parasites/mL			Excluding 6 and 60 parasites/mL			Specificity^b^
Laboratory	#positive (%)	n^a^	Sensitivity	#positive (%)	n^a^	Sensitivity	
**UW**	36 (72%)	50	90.0%	30 (75%)	40	100.0%	100.0%
**Oxford**	34 (68%)	50	85.0%	30 (75%)	40	100.0%	100.0%
**NIH**	66 (66%)	100	82.5%	60 (75%)	80	100.0%	100.0%
**RUMC**	32 (64%)	50	80.0%	30 (75%)	40	100.0%	100.0%
**Maryland**	35 (70%)	50	87.5%	29 (73%)	40	96.7%	100.0%

a The total number of specimens tested, including parasite-negative specimens.

b Based on 10 parasite-negative specimens per laboratory (20 at NIH).

Analytical sensitivity was below 100% at the 60 parasites/mL level: UW detected 6/10 samples, Oxford 4/10, NIH 6/20, Maryland 4/10 and RUMC 2/10. One positive sample was detected by the UW laboratory amongst the 6 parasites/mL samples at a concentration consistent with the presence of a single parasite in the sample and consistent with the overall frequency of positives at the 60 parasites/mL level as well. The ‘trace’ (6 parasites/mL) specimens were not included in estimates of sensitivity and specificity because they cannot be categorically considered positive for the following assay-specific reasons. For the low volume RT-PCR assay at UW, most of these samples are negative because a density of 6 parasites/mL equates to less than one parasite per sample on average. Nonetheless, when samples at this level are positive by RT-PCR due to an intact parasite, the parasite contains ∼3500 copies of the targeted 18S rRNA. For higher volume DNA assays, the lack of positive results at the 6 parasites/mL level reflects a combination of similar Poisson distribution limitations (e.g., 6 parasites/mL is an average of 1.2 parasites/sample for 200 µL samples or 3 parasites/sample for 500 µL samples) as well as limitations on PCR performance at low copy numbers (e.g., 2–10 DNA copies/sample). Thus, the virtual absence of positive results in samples at 6 parasites/mL was expected, indicates that positive results are due to intact parasites and not free template and supports the high specificity shown by all laboratories. False negative results at the 60 parasites/mL level can be attributed to the presence of multiply-infected RBCs that result in a more heterogeneous distribution of parasites upon dilution to low parasitemia than would be expected if virtually no multiply-infected cells were present. A related type of heterogeneity has also reported for malaria microscopy – in thick blood smears, malaria parasites are sometimes unevenly distributed (known as ‘grouping’) [60]. At low parasite densities, grouping results in a few microscopic fields containing more parasites than expected while most fields contain fewer or zero parasites, leading to overdispersion of the Poisson distribution [61]. Much like ‘grouping’ in thick blood smears, multiply-infected cells contain a larger-than-expected proportion of the NAT target(s), thereby altering the frequency of parasite-containing samples at low parasitemias. Nonetheless, the overall data indicate agreement between laboratories across a wide range of parasite densities, with gradual loss of positivity as predicted by assay LoDs.

Differences from the expected values (quantitative bias) were assessed using correlation and Bland-Altman (difference) plots, which are summarized in **Table 3**. In general, all assays behaved linearly (slope ∼1.0 and r^2^>0.9), indicating comparable template amplification efficiencies. The average quantitative bias across all laboratories was <±0.18 log_10_ parasites/mL compared to expected values, with the exception of the Oxford qPCR. In addition, most laboratories maintained a 95% confidence range within ±0.5 log_10_ parasites/mL compared to the expected values with the exception of Maryland whose assay had lower limit of the 95% confidence interval extending to −0.66 log_10_ parasites/mL. The Oxford qPCR showed an average difference of −0.54 log_10_ parasites/mL (95%CI −0.90 to −0.19 log_10_ parasites/mL), thereby underestimating relative to expected values and other laboratories. This difference was consistent across the range of parasite densities for Oxford and the confidence interval for the Oxford qPCR was within ±0.5 log_10_ parasites/mL of the average Oxford values.

**Table 3:** Correlation and agreement between assay-derived data and expected values.

	Correlation^a^		Agreement^b^	
Laboratory	Slope	r^2^	Quantitative bias (95% confidence interval)	n^c^
**UW**	1.04	0.98	−0.05 (−0.39–0.29)	37
**Oxford**	1.00	0.98	−0.54 (−0.90–−0.19)	34
**NIH** d	0.97	0.97	0.13 (−0.30–0.55)	66
**RUMC**	0.90	0.99	0.1 (−0.29–0.48)	32
**Maryland**	0.99	0.96	−0.18 (−0.66–0.31)	35

a Each laboratory's assay-derived data were plotted against the expected values, and the slope (Δ assay-derived value/Δ expected value) and coefficient of determination (r^2^) were calculated using Microsoft Excel. A slope of 1.0 and r^2^ value of 1.0 indicates perfect correlation.

b Bland-Altman difference plots were used to calculate the mean quantitative bias as the mean of the differences between each reported value and its expected value. Values are in log_10_ parasites/mL. An absolute value of ≤0.5 log_10_ parasites/mL indicates an absence of quantitative bias.

c All calculations were based on all NAT-positive samples.

d NIH quantities were generated by regression of C_T_ values to nominal values and should be viewed as a measure of variation only.

Precision (%CV) was evaluated for each laboratory's assay for the high, mid and low parasite density samples (**Table 4**). As expected, the highest parasite density samples showed the lowest degree of variation and, with the exception of the Maryland qPCR, the lowest parasite density samples had the highest variation. Variation was generally <10%CV at all levels. This level of precision is considered acceptable by most validation criteria and is likely to be more than adequate for purposes of modeling parasite growth dynamics [42], [43]. Very low parasite density samples were not included in this analysis because of the smaller number of positive samples.

**Table 4:** Precision statistics by laboratory.

	High (300,000 parasites/mL)		Mid (6,000 parasites/mL)		Low (600 parasites/mL)	
Laboratory	%CV	n	%CV	n	%CV	n
**UW**	1.5	10	1.6	10	5.3	10
**Oxford**	3.5	10	3.3	10	7.1	10
**NIH** a	1.7	20	2.6	20	10.0	20
**RUMC**	0.9	10	4.5	10	6.9	10
**Maryland**	3.4	10	11.1	10	5.1	9

a NIH quantities were generated by regression of C_T_ values to nominal values and should be viewed as a measure of variation only.

### Root cause analyses of differences

Two of the network groups further investigated apparently aberrant results to determine if the root cause could be identified. The single false negative 600 parasites/mL sample at the University of Maryland was found to be qualitatively positive, but at a concentration below the quantitative LoD established for that assay. Root cause analysis showed that the DNA extraction control used to monitor extraction efficiency was lower than the expected value, indicating that poor sample extraction was at fault - re-extraction of the specific sample was not possible since the material was completely consumed in the initial testing.

Additional studies were conducted by Oxford University to estimate the contribution of blood filtering and calibrator matrix differences to the observed quantitative shift. To test the hypothesis that parasites may be lost during blood filtration, parasites diluted in pre-leukocyte-depleted blood were tested by Oxford qPCR either with or without filtration. Filtration reduced the measured parasite density by 13–57% (−0.06 to −0.37 log_10_-fold) (**Figure S1**). To determine if the matrix used to dilute the plasmid DNA calibrators at Oxford also contributed to the quantitative shift, DNA extracted from malaria-negative volunteers was used in lieu of water to dilute the plasmid DNA calibrator in the qPCR reaction. One-hundred fifty copies of plasmid DNA were added since this corresponds to the amount of plasmid DNA equal to 1000 parasites/mL by the Oxford qPCR assay. In the presence of the negative blood matrix, the Oxford qPCR C_T_ was delayed by a median of 1.1 cycles as compared to the previously used water matrix – this difference was consistently observed using blood from three different donors (range 1.1–1.4 cycles) (**Figure S1**). This C_T_ shift corresponds to a 58–65% reduction in the apparent parasite density (median −0.39 log_10_-fold, range −0.37 to −0.46 log_10_-fold) relative to amplification using a water-only diluent reduction (**Figure S1**).

## Discussion

This study represents the first major malaria NAT EQA exercise to our knowledge amongst CHMI centers providing assay-to-assay comparisons that attempt to fully account for all variables contributing to assay performance. The data indicate that CHMI centers listed here are reporting comparable results. CHMI studies seek to ensure the safety of the trial participants and the integrity of the trial data. In this regard, all centers achieved expected analytical sensitivities based on known LoDs. Based on clinical trial data and supported by modeling studies [41]–[43], the assays compared here are likely to become positive before patent parasitemia develops following CHMI with five mosquito bites. No false positive results were reported for any of the 10–20 blinded malaria-negative samples sent to each laboratory. Quantitative variation was <10%CV amongst the high, mid and low parasite density samples. The average quantitative bias across all laboratories was <±0.18 log_10_ parasites/mL compared to expected values, and all laboratories except for Oxford showed a 95% confidence range within ±0.5 log_10_ parasites/mL compared to the expected values.

The Oxford assay was highly sensitive, specific and precise, but was quantitatively shifted compared to other assays and to expected results. As described in the Results, additional studies conducted by Oxford University determined that both parasite losses due to Whatman VFE filtering of whole blood and differences in the matrix used for plasmid DNA calibrators at Oxford contributed to this shift. This is the first report to our knowledge that demonstrates parasite losses due to the filtering step. This step was added at Oxford to remove co-purified leukocyte genomic DNA (gDNA), which may inhibit some PCR assays [62]. Thus, while filtration removes inhibitors like gDNA, it also removes parasites. In addition, the Oxford plasmid calibrators are normally diluted in a water matrix, which lacks additional PCR inhibitors common to whole blood extractions (e.g., immunoglobulin G, hemoglobin and lactoferrin [63], [64]). Since the water-diluted plasmid calibrator was detected earlier than when diluted in a whole blood matrix, it appears that PCR inhibitors present in the eluates obtained from blood delay PCR target amplification and also contribute to the quantitative reduction in parasites when a water-diluted standard curve was used. This type of matrix effect has been reported in biological samples [65]. Despite the shift in absolute quantitation, relative quantitation between samples using the Oxford assay was comparable to that of other centers. The Oxford qPCR is reporting positive and negative results in complete agreement with other laboratories, even at the low 60 parasites/mL level suggesting that the combined effects of blood filtration and the different calibrator matrix did not markedly change the overall qualitative results. Nonetheless, re-calibration of the Oxford qPCR method that will account for these differences is underway. This EQA effort helped to identify an easily fixed methodological difference in calibration that may account for differences in absolute quantification, and work is underway at Oxford to rectify this shift. Overall, the results of most laboratories were within the expected quantitative range, and these findings should be reassuring to vaccine and drug makers, sponsors and regulators as NAT gradually replaces microscopy for safety and efficacy endpoints.

With the plethora of malaria NAT methods reported in the literature, quality indicators are needed to help select and maintain methods suitable for use in CHMI studies. However, malaria NAT EQA and consensus malaria NAT guidelines do not yet exist in part because over 65 different assays have been reported for NAT-based malaria diagnosis. The literature includes many variations on extraction, amplification and detection, including single-step and nested electrophoresis-based PCR, qPCR, qRT-PCR and nucleic acid-based sequence amplification (NASBA) with an even wider variety of reported primer and probe combinations (briefly reviewed in [22]). Additional novel assays continue to be developed, including potentially field-friendly approaches such as loop-mediated isothermal amplification [66]. While numerous other gene targets are reported, the most widely used *Plasmodium* NAT targets are sequences within the developmentally-regulated 18S rRNAs (by RT-PCR or NASBA) or their coding genes (by PCR) [67]. However, 18S rRNAs and their corresponding coding genes are not uniformly captured by a single set of reagents (primers and probe), and assay design can greatly alter sensitivity and specificity as recently reviewed [22]. RT-PCR tests target A-type (asexual stage) 18S rRNAs to take advantage of the fact that A-type (but not sexual-stage S-type) 18S rRNAs are biologically amplified to ∼3500 copies per ring-stage parasite during the red blood cell stage of infection [41]. PCR tests can target any of the 18S rRNA-coding genes, and some primers and/or probes are designed to capture sequences shared by more than one of the genes (up to five including one pseudogene) thereby incrementally increasing assay sensitivity. Thus, RT-PCR assays become positive earlier in the amplification process (lower cycle threshold) compared to PCR, and this can improve RT-PCR sensitivity particularly with small sample volumes and/or extremely low parasite densities (S. Murphy, unpublished data). While rRNA copies are more abundant than the parent genes, samples for RT-PCR must be preserved by adding a stabilizing buffer or by making dried blood spots at the time of collection [58], whereas samples for PCR can be simply frozen. These aspects of malaria NAT have mostly been studied using laboratory strains of *P. falciparum*, and future work will need to address assays designed to test for other human *Plasmodium* spp. since species and sub-species diversity in field settings affects assay performance [68], [69].

Thus, because of the diversity of pre-analytical processing, extraction and amplification techniques, a single EQA sample type cannot currently be used in all assays. EQA efforts must therefore account for these methodological variations in pre-analytical (collection, stabilization) and analytical (extraction, amplification) steps.

To support clinical trials using malaria NAT, a formal EQA program is needed. Some previous efforts have been made to improve the quality of malaria NAT, although none provide formal EQA. As mentioned above, the WHO previously developed and distributed its single concentration standard to many laboratories for characterization purposes [52], however, this standard is unsuitable for RNA-based assays and does not precisely mimic clinical samples. Another group compared PCR primers and probes used by several centers but it was not possible for the investigators to perform all the pre-analytical, analytical (extraction, amplification and detection) and post-analytical steps for each assay as originally described and therefore the study did not assess overall assay performance [53]. Most recently, a study of field samples in Brazil compared two conventional PCR methods against microscopy and concluded that the PCR protocols showed low reproducibility at sub-microscopic densities [70]. In contrast, the groups tested in our EQA study generated reproducible data at sub-microscopic densities (e.g., 600 parasites/mL), but our study was performed using laboratory-generated samples so this may change when field samples are used. True EQA comparisons, such as the work described herein, are needed to fully account for all factors that lead to assay variability and ensure that high quality assays are in place to support clinical and field studies.

To continue our work and fulfill the need for an ongoing EQA program, we propose creation of the Molecular Malaria Quality Assessment (MolMalQA) Program as a multi-lateral effort involving one or more core reference laboratories and a larger network of partner laboratories at centers performing malaria clinical trials, drug efficacy testing and surveillance activities. The core laboratories would provide malaria EQA samples, produce an international calibrator suitable for use in DNA and RNA assays and pursue harmonization activities in consultation with network partners. The malaria effort will be somewhat more complicated than EQA programs for many viral pathogens because of the complexity of the parasite lifecycle and the diversity of available tests. At a minimum, it will be necessary to provide EQA samples for DNA- and RNA-based assays alike and to support both liquid and dried blood spot formats. For global EQA, we propose to use synchronized, cultured ring-stage malaria parasites diluted into whole blood since this mimics clinical samples. The EQA program will need to develop whole blood panels derived from a spectrum of healthy human subjects with diverse blood types to ensure that results are not skewed by a single blood source. In future, gametocytes, the sexual malaria stage parasite found in humans, can be provided as EQA samples if needed. The EQA program can also undertake stability studies to assess the collection, transport and long-term storage stability of samples intended for malaria DNA and RNA testing to determine if the relatively greater stability of DNA outweighs the increased abundance of RNA for use in large field studies.

Eventually, assays in use should also be harmonized around a single or very few high-quality diagnostic protocols. Selection of such assays could be made by reviewing EQA data and studying protocols and instrument requirements in consultation with the network of CHMI centers.

In addition to ensuring valid diagnostic data in clinical trials, an EQA program would also aid in NAT surveillance for the malaria elimination/eradication agenda. The relevance of malaria NAT EQA for surveillance efforts should not be overlooked because global success in reducing malaria incidence will reach a point at which control and eradication decisions will require monitoring of infection among individuals harboring parasites at densities beneath the LoD of both microscopy and RDTs [71].

To more closely align quantitative results across centers and eliminate lot-to-lot variation in cultured parasites, we further advocate for development of synthetic nucleic acid sequences diluted in whole blood for use as absolute calibrators. Such reagents allow determination of exact target copy numbers, which can be translated to parasite densities by testing parasite-containing controls against a calibrator standard curve. However, since the 18S rRNA targets vary between testing centers, no single naturally-occurring target sequence is shared by all laboratories. Some centers target the asexual-type 18S rRNAs or the coding genes while others target the genes encoding the sexual-type 18S rRNAs. Thus, cloning just one of these genes fails to capture other laboratories' targets. Novel synthetic sequences are therefore needed to provide cross-network calibrators suitable for more than one assay - such materials are in development (S. Murphy, unpublished data).

In summary, malaria NAT EQA will help safeguard the reliability and comparability of data produced in clinical trials by CHMI centers and will support future extended use of malaria NATs in other contexts. Through collaboration and with multi-lateral funding, a formal EQA program can be developed and implemented worldwide for the benefit of the malaria field and those it serves.

## Supporting Information

Figure S1 **Parasite loss due to filtering and calibrator matrix differences contribute to the quantitative shift in Oxford qPCR.** A: Filtration using the Whatman VFE plate results in loss of parasites. Cultured parasites were combined with leukocyte-depleted blood and then filtered or not as indicated in the figure. Results of Oxford qPCR are shown; each point represents the mean of triplicate PCR wells. B-C: Oxford qPCR in a whole blood matrix results in delayed C_T_ and lower apparent parasite density than when a water matrix is used. Eluates from leukocyte-depleted blood or a water control were added to qPCR reactions containing 150 copies of a plasmid DNA calibrator. Panel B depicts C_T_ values. Panel C shows the apparent parasite density; the horizontal dashed line represents the result in the presence of water-only diluent (150 plasmid copies/reaction  = 1000 parasites/mL in the Oxford assay by definition). Each point in B-C represents the result in an individual PCR well.
(TIF)
