# Supplementary figures and images for: External Quality Assurance of Malaria Nucleic Acid Testing for Clinical Trials and Eradication Surveillance
Source: PLoS One. 2014 May 16;9(5):e97398. doi: 10.1371/journal.pone.0097398 (PMC4023973; doi:10.1371/journal.pone.0097398)

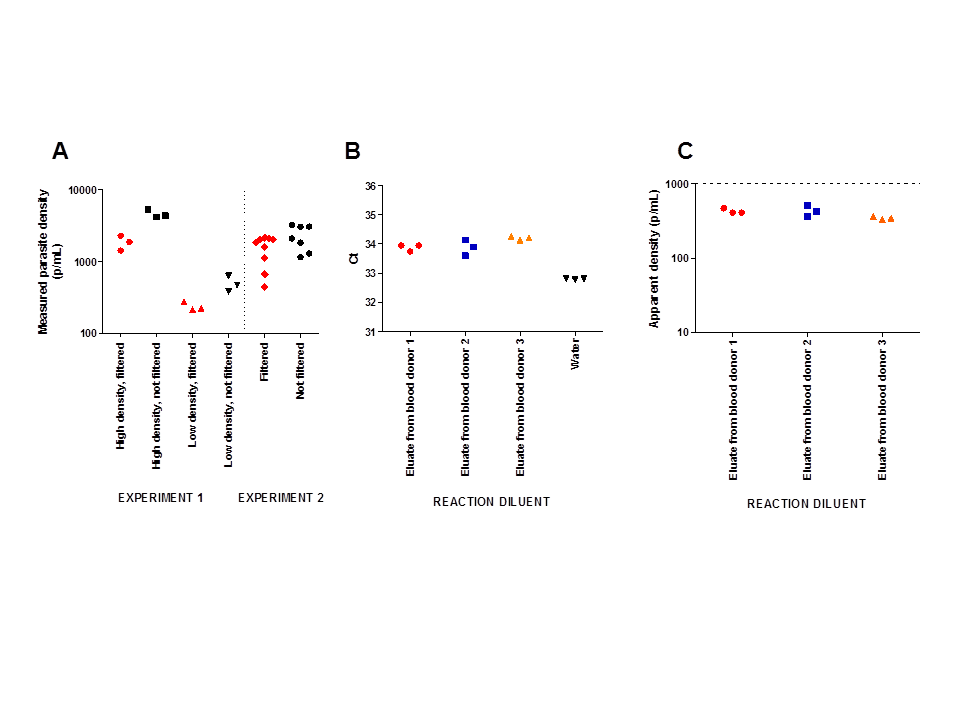

Supplement: Figure S1 — Parasite loss due to filtering and calibrator matrix differences contribute to the quantitative shift in Oxford qPCR. A: Filtration using the Whatman VFE plate results in loss of parasites. Cultured parasites were combined with leukocyte-depleted blood and then filtered or not as indicated in the figure. Results of Oxford qPCR are shown; each point represents the mean of triplicate PCR wells. B-C: Oxford qPCR in a whole blood matrix results in delayed CT and lower apparent parasite density than when a water matrix is used. Eluates from leukocyte-depleted blood or a water control were added to qPCR reactions containing 150 copies of a plasmid DNA calibrator. Panel B depicts CT values. Panel C shows the apparent parasite density; the horizontal dashed line represents the result in the presence of water-only diluent (150 plasmid copies/reaction = 1000 parasites/mL in the Oxford assay by definition). Each point in B-C represents the result in an individual PCR well. (TIF) [file pone.0097398.s001.tif]
